# Characteristics of mortal COVID-19 cases compared to the survivors

**DOI:** 10.18632/aging.202216

**Published:** 2020-11-21

**Authors:** Xianghui Zhou, Zhipeng Cheng, Dan Shu, Wenyi Lin, Zhangyin Ming, Wei Chen, Yu Hu

**Affiliations:** 1 Department of Hematology, Union Hospital, Tongji Medical College, Huazhong University of Science and Technology, Wuhan 430022, China; 2 Collaborative Innovation Center of Hematology, Huazhong University of Science and Technology, Wuhan 430022, Hubei, China; 3 Department of Pharmacology, School of Basic Medicine, Tongji Medical College of Huazhong University of Science and Technology, Wuhan 430022, China; 4 Tongji-Rongcheng Center for Biomedicine, Huazhong University of Science and Technology, Wuhan 430022, China; 5 Hubei Clinical Medical Center of Cell Therapy for Neoplastic Disease, Wuhan 430022, Hubei, China; 6 Laboratory of Vaccine and Antibody Engineering, Beijing Institute of Biotechnology, Beijing 100071, China

**Keywords:** coronavirus disease 2019, COVID-19, SARS-CoV-2, death cases, survival cases

## Abstract

The outbreak of coronavirus disease 2019 (COVID-19) initially occurred in December 2019 and triggered a public health emergency. The increasing number of deaths due to this disease was of great concern. Therefore, our study aimed to explore risk factors associated with COVID-19 deaths.

After having searched the PubMed, EMBASE, and CNKI for studies published as of August 10, 2020, we selected articles and extracted data. The meta-analysis was performed using Stata 16.0 software.

Nineteen studies were used in our meta-analysis. The proportions of comorbidities such as diabetes, hypertension, malignancies, chronic obstructive pulmonary disease, cardio-cerebrovascular disease, and chronic liver disease were statistically significantly higher in mortal COVID-19 cases. Coagulation and inflammatory markers, such as platelet count, D-dimer, prothrombin time, C-reactive protein, procalcitonin, and interleukin 6, predicted the deterioration of the disease. In addition, extracorporeal membrane oxygenation and mechanical ventilation predicted the poor prognosis during its progression.

The COVID-19 pandemic is still evolving, placing a huge burden on healthcare facilities. Certain coagulation indicators, inflammatory indicators, and comorbidities contribute to the prognosis of patients. Our study results may help clinicians optimize the treatment and ultimately reduce the mortality rate.

## INTRODUCTION

In late December 2019, a cluster of cases of “pneumonia of unknown cause” were reported in Wuhan, China. These patients were epidemiologically linked to a seafood and wildlife wholesale market. The initial diagnosis was pneumonia of an unknown etiology [1]. The National Health Commission of the People’s Republic of China announced that a novel coronavirus, which is now renamed as severe acute respiratory syndrome coronavirus 2 (SARS-CoV-2) by the World Health Organization, was responsible for the global outbreak [2]. This causative pathogen SARS-CoV-2 has been identified as a novel enveloped RNA beta coronavirus [3]. Coronaviruses (CoVs) primarily target the human respiratory system and cause severe acute respiratory syndrome (SARS) and the Middle East respiratory syndrome (MERS). More importantly, Coronavirus disease (COVID-19) shares many similar clinical symptoms with SARS-CoV and MERS-CoV [2]. For instance, infected patients predominantly present with fever, cough, and radiological ground-glass lung opacities [4]. SARS-CoV-2 has gradually spread worldwide, and the disease caused by this virus, named COVID-19, is now a pandemic. The rapid increase in the transmission of this virus is mainly due to direct contact with infected people through respiratory droplets and touching contaminated objects. Moreover, the virus can be spread by asymptomatic carriers [5]. As of August 14, 2020, 20,730,456 laboratory-confirmed cases and 751,154 deaths in 216 countries, regions, or territories have been documented [https://www.who.int (accessed 14 August 2020)].

Since the outbreak, several medical centers worldwide have collected data (epidemiological data, clinical patient data etc.) on relevant cases, and medical teams have focused their efforts into finding a treatment for COVID-19. Many studies have been published in a timely manner to provide the public with a real-time understanding of COVID-19. China is one of the main epidemic areas of COVID-19, from where some clinical data have been summarized. Certain studies also determined that an updated analysis of cases throughout mainland China could help identify the defining clinical characteristics and severity of COVID-19. A previous study showed that 7,736 COVID-19 patients had been hospitalized in 30 provinces, autonomous regions, or municipalities across China as of January 29, 2020 [6]. The median age of these patients was 47 years, and fever and cough were the most common symptoms. On admission, ground-glass opacity was the most common radiologic finding on chest computed tomography (CT) (56.4%). No radiographic or CT abnormality was found in 157 of 877 patients (17.9%). Clinical data from hospitals nationwide are gradually being published. Some retrospective studies investigated the specificity and sensitivity of imaging, nucleic acid detection, feasibility of multiple treatments (e.g., convalescent plasma therapy and antiviral therapy), and clinical characteristics of both critical and non-critical patients. These clinical characteristics were compared between mortal and survival cases [7–10]. Besides, several systematic reviews have summarized such comparisons (e.g., imaging findings, laboratory test results, and clinical symptoms). However, only limited data on clinical characteristics of mortal cases and treatment outcomes of critical COVID-19 cases are available because of the relatively small numbers of non-survivors in previous studies. Very few systematic reviews of mortality risk factors in COVID-19 patients have been conducted, although they are of great importance to reduce mortality.

As COVID-19-related mortality continues to grow worldwide, increasing attention has been paid to summarizing the findings and complications obtained from clinical signs, laboratory and chest CT, and treatments that mortal cases received. We conducted this meta-analysis to provide a comprehensive understanding of the characteristics of COVID-19-related deaths and compare them with those of survivors to enable better investigations of the prognostic factors of COVID-19 in infected individuals. Our study results are expected to provide evidence on specific risk factors of COVID-19 and aid in reducing its associated fatality.

## RESULTS

### Literature search and screening

**Figure 1:**
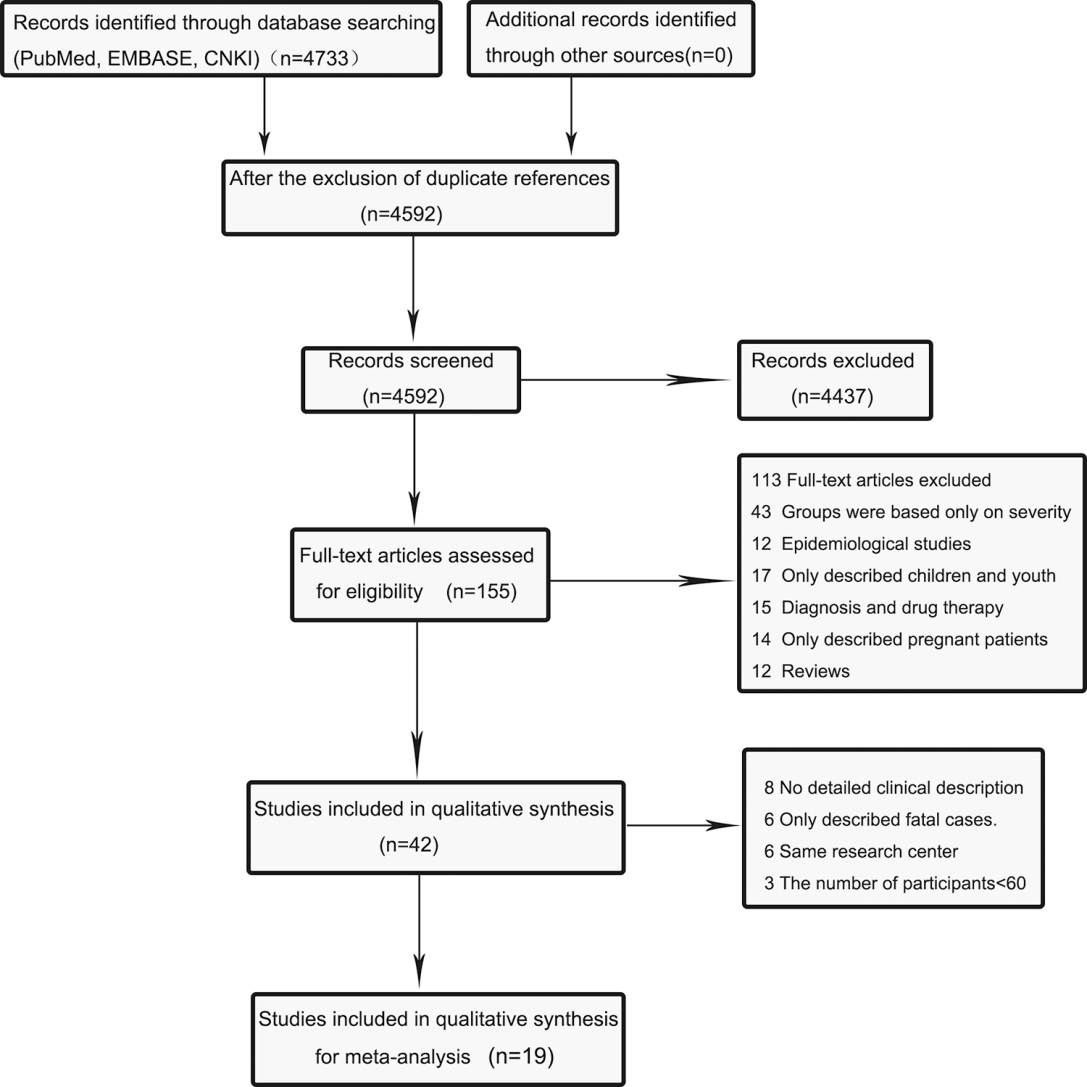
**Flow diagram of the literature search and selection process in the meta-analysis.**

The database searches identified a total of 4,896 potentially relevant articles, including 4375 in PubMed, 298 in EMBASE, and 60 in CNKI. Of these articles, 141 were excluded due to duplication. After screening titles and abstracts, we further excluded 4437 due to non-relevance. After full texts were carefully reviewed, 113 articles were removed for not reporting clinical features of COVID-19 or describing death cases, or for some other reasons (Supplementary File 1). Forty-two articles were included in the meta-analysis, of which 8 articles did not provide a detailed clinical description of death cases and cured ones, and 6 articles only described fatal cases. Besides, 6 articles reported a study conducted in the same medical center, and 3 articles had sample sizes of <60 participants. Finally, the meta-analysis included 19 eligible articles [11–29]. The flow diagram (Figure 1) illustrates the detailed procedure of literature search.

### Characteristics of studies and demographic features

Of 19 studies included in the meta-analysis, some were conducted in Asia, some in Europe, and the others in the Americas. Their sample sizes varied between 100 and 7,371 participants (Table 1). These studies showed that the proportion of male was significantly higher in the death group than that in the survival group (odds ratio [OR]=1.68, 95% confidence interval [CI]: 1.36-2.08, *P*<0.001). Furthermore, the mean age of the death group ranged from 65.6 to 78.7 years, compared to 46-68.7 years in the survival group. According to the meta-analysis results, the death group had an older mean age than the survival group (mean difference [MD]=12.38, 95% CI: 10.82-13.95, *P*<0.001). Considering the high heterogeneity of age and gender estimates across 19 studies, we used the random-effects model (Tables 2, 3 and Supplementary Figure 1).

**Table 1:** Main characteristics of the included studies in our-analysis.

**Study**	**Journal**	**Date**	**Country**	**Sample size**			**Male:N(%)**		**Age:mean±SD**	
				**Total**	**Survivor**	**Non-survivor**	**Survivor**	**Non-survivor**	**Survivor**	**Non-survivor**
Cao,J.L.	Clin Infect Dis	2020/4/3	China	102	85	17	40(47%)	13(76%)	55.3±14.3	72±13.6
Du, R. H.	Eur Respir J	2020/4/10	China	179	158	21	87 (55%)	10 (48%)	56.0±13.5	70.2±7.7
Wang, L.	J Infect	2020/4/3	China	339	274	65	127(46%)	39(60%)	68.7±7.5	76.3±9.9
Javanian, M.	Rom J Intern Med	2020/5/13	Iran	100	81	19	39 (49%)	12 (57%)	57.7±13.6	69.3±11.1
Li, Q.B.	Leukemia	2020/6/13	China	1449	1327	122	643(48%)	90(74%)	53.7±17.8	70±11.3
Okoh, A. K.	Int J Equity Health	2020/6/12	USA	251	154	97	69 (45%)	60 (62%)	57.7±17.2	67±15.8
Rath, D.	Clin Res Cardiol	2020/6/17	Germany	123	107	16	65 (61%)	12 (75%)	67 ± 15	73 ± 16
Khalil, K.	J Infect	2020/6/21	United Kingdom	217	158	59	87 (55%)	42 (71%)	64.0±2.6	76.1±3.3
Nowak, B.	Pol Arch Intern Med	2020/5/19	Poland	169	123	46	57 (46%)	30 (65%)	59.3± 20.1	75.3 ±11.9
Shahriarirad, R.	BMC Infect Dis	2020/6/20	Iran	113	104	9	66 (93%)	5 (7%)	/	/
Wang, K.	Clin Infect Dis	2020/5/4	China	296	277	19	129 (47%)	11 (58%)	46.0 ± 14.4	65.6 ± 12.6
Aloisio, E.	Arch Pathol Lab Med	2020/7/11	Italy	427	338	89	223(66.0%)	70(78.7%)	58.3±15.6	73.3±9.8
Baqui, P.	Lancet Glob Health	2020/7/6	Brazil	7371	4043	3328	2300(56.9%)	1990(59.8%)	51.6±17.0	65.6±15.9
Berenguer, J.	Clin Microbiol Infect	2020/8/8	Spain	4035	2904	1131	1666(57.4%)	767(67.8%)	63.7±17.8	78.7±11.1
Bonetti, G.	Clin Chem Lab Med	2020/6/24	Italy	144	74	70	51(68.9%)	45(64.3%)	62.6±15.0	75.4±15.0
Gayam, V.	J Med Virol	2020/7/17	USA	408	276	132	155(56.2%)	76(57.6%)	63±14.9	71±13.5
Liu, J.	Ann Intensive Care	2020/8/2	China	1190	1033	157	535(51.8%)	100(63.7%)	55.7±14.1	69.3±11.2
Salacup, G.	J Med Virol	2020/7/4	USA	242	190	52	96(50.5%)	27(51.9%)	64.1±15.1	73.2±11.0
Yu, C.Z.	Am J Prev Med	2020/6/23	China	1464	1252	212	586(46.8%)	150(70.8%)	60.5±15.6	69.5±10.8

**Table 2:** Results of meta-analysis (binary variable).

**Variable**	**No. of Studies**	**OR**	**95%CI**	**P-Value**	**Heterogeneity**		**Model**
					**I^2^**	**p**	
Gender-male	19	1.68	1.36,2.08	<0.001	78.50%	0.00	Random
China	7	2.25	1.55,3.25	<0.001	66.18%	0.01	Random
Others	12	1.40	1.16,1.68	<0.001	57.37%	0.00	Random
Fever	11	0.84	0.57,1.21	0.34	59.37%	0.01	Random
China	6	0.95	0.51,1.77	0.87	69.55%	0.01	Random
Others	5	0.71	0.52,0.98	0.50	0.00%	0.04	Fixed
Cough	12	0.87	0.56,1.35	0.54	81.45%	0.00	Random
China	7	0.99	0.51,1.90	0.97	87.51%	0.00	Random
Others	5	0.79	0.58,1.08	0.14	42.06%	0.14	Fixed
Sputum	6	1.30	1.04,1.60	0.02	45.00%	0.11	Fixed
Headache	8	0.75	0.40,1.42	0.38	49.57%	0.06	Random
China	5	0.68	0.24,1.95	0.47	67.86%	0.02	Random
Others	3	0.76	0.38,1.51	0.42	0.00%	0.37	Fixed
Myalgiaor	9	0.94	0.73,1.23	0.68	6.21%	0.38	Fixed
Fatigue	9	1.65	1.01,2.66	0.04	85.99%	0.00	Random
China	5	1.84	0.89,3.78	0.10	92.85%	0.00	Random
Others	4	1.21	0.85,1.70	0.30	0.00%	0.67	Fixed
Diarrhoea	8	0.68	0.39,1.19	0.18	50.32%	0.07	Random
China	4	0.91	0.63,1.35	0.66	8.01%	0.35	Fixed
Others	4	0.34	0.16,0.75	0.01	39.66%	0.17	Fixed
Haemoptisis	5	1.40	0.66,2.97	0.38	0.00%	0.72	Fixed
Nausea/Vomiting	7	0.83	0.52,1.34	0.44	37.64%	0.14	Fixed
Sore throat	6	0.61	0.35,1.06	0.08	0.00%	0.92	Fixed
Dyspnoea	7	1.88	1.16,3.03	0.01	66.89%	0.01	Random
China	4	2.53	1.39,4.62	<0.001	73.42%	0.02	Random
Others	3	1.11	0.65,1.86	0.72	0.00%	0.83	Fixed
Diabetes	18	2.51	1.86,3.35	<0.001	87.32%	0.00	Random
China	6	2.92	1.80,4.76	<0.001	67.63%	0.02	Random
Others	12	2.32	1.60,3.39	<0.001	90.48%	0.02	Random
Hypertension	15	2.39	1.95,2.89	<0.001	48.54%	0.00	Random
China	5	2.80	1.72,4.57	<0.001	76.19%	0.03	Random
Others	10	2.27	1.77,2.94	<0.001	48.20%	0.02	Random
Malignancies	12	2.36	1.68,3.29	<0.001	0.00%	0.90	Fixed
COPD	11	1.99	1.51,2.64	<0.001	30.06%	0.16	Fixed
Cardiovascular disease	17	2.92	2.08,4.10	<0.001	89.78%	0.00	Random
China	5	4.01	1.82,8.76	<0.001	81.84%	0.00	Random
Others	12	2.66	1.79,3.97	<0.001	91.60%	0.00	Random
Cerebrovascular disease	10	2.69	2.01,3.60	<0.001	20.37%	0.26	Random
Chronic renal disease	15	3.03	1.77,5.16	<0.001	80.83%	0.00	Random
China	5	1.75	0.98,3.13	0.06	36.58%	0.18	Fixed
Others	10	3.46	1.80,6.69	<0.001	85.39%	0.00	Random
Chronic liver disease	9	2.14	1.60,2.86	<0.001	17.78%	0.28	Fixed
Ground Glass Opacity	5	0.54	0.43,0.69	<0.001	44.01%	0.13	Fixed
Consolidation	4	1.80	1.39,2.34	<0.001	19.37%	0.29	Fixed
Acute cardiac injury	4	16.44	6.23,43.82	<0.001	82.67%	0.00	Random
Acute kidney injury	4	24.53	4.85,125.21	<0.001	90.12%	0.00	Random
ARDS	5	70.81	23.81,210.61	<0.001	81.03%	0.00	Random
China	3	179.47	109.95,292.95	<0.001	21.65%	0.28	Random
Others	2	18.54	8.25,41.68	<0.001	0.00%	0.85	Fixed
ECMO	3	28.50	5.93,135.64	<0.001	0.00%	0.65	Fixed
Noninvasive ventilation	6	11.25	1.80,70.11	0.01	89.92%	0.00	Random
China	3	64.07	14.44,284.29	<0.001	83.15%	0.03	Random
Others	3	1.63	0.43,6.23	0.47	0.00%	0.78	Fixed
Invasive mechanical ventilation	6	24.29	5.53,106.70	<0.001	91.36%	0.00	Random
China	3	81.45	46.06,144.03	<0.001	0.00%,	0.38	Fixed
Others	3	5.05	1.15,22.42	0.03	78.20%	0.03	Random

**Table 3:** Results of meta-analysis (continuous variable).

**Variable**	**No. of Studies**	**Mean Diff.**	**95%CI**	**P-Value**	**Heterogeneity**		**Model**
					**I^2^**	**p**	
Age	18	12.38	10.82,13.95	<0.001	87.27%	0.00	Random
China	7	13.18	9.94,16.42	<0.001	84.76%	0.00	Random
Others	11	12.20	10.46,13.93	<0.001	85.88%	0.00	Random
haemoglobin	9	-1.40	-5.10,2.31	0.46	86.81%	0.00	Random
China	3	1.16	-1.82,4.14	0.45	61.92%	0.06	Random
Others	6	-3.32	-8.83,2.18	0.24	80.30%	0.00	Random
platelet count	9	-35.30	-58.11,-12.50	<0.001	93.93%	0.00	Random
China	3	-66.29	-102.31,-30.27	<0.001	93.41%	0.00	Random
Others	6	-11.87	-16.02,-7.72	<0.001	0.00%	0.89	Fixed
D-dimer	12	5.39	1.32,9.46	0.01	99.97%	0.00	Random
China	5	7.82	-0.88,16.53	0.08	99.95%	0.00	Random
Others	7	3.60	0.19,7.02	0.04	99.81%	0.00	Random
prothrombin time	6	1.32	0.42,2.21	<0.001	97.08%	0.00	Random
APTT	8	1.19	-0.34,2.72	0.13	89.28%	0.00	Random
China	5	1.56	-0.58,3.70	0.15	92.05%	0.00	Random
Others	3	0.56	-1.26,2.38	0.55	63.63%	0.06	Random
CRP	13	63.81	38.14,89.49	<0.001	98.82%	0.00	Random
China	4	75.58	38.05,113.12	<0.001	98.36%	0.00	Random
Others	9	58.49	24.50,92.49	<0.001	98.55%	0.00	Random
Procalcitonin	7	0.64	0.22,1.06	<0.001	99.88%	0.00	Random
China	3	0.26	0.11,0.41	<0.001	94.24%	0.00	Random
Others	4	0.96	0.33,1.59	<0.001	95.41%	0.00	Random
Interleukin 6	5	171.45	66.37,276.53	<0.001	99.47%	0.00	Random
China	3	209.35	43.43,375.26	0.01	99.46%	0.00	Random
Others	2	119.2	-4.08,242.48	0.06	99.28%	0.00	Random

### Clinical symptoms

According to the meta-analysis of included studies, the death group had statistically significant higher proportions of patients with sputum (OR=1.30, 95% CI: 1.04-1.60, *P*=0.02), fatigue (OR=1.65, 95% CI: 1.01-2.66, *P*=0.04), and dyspnea (OR=1.88, 95% CI: 1.16-3.03, *P*=0.01) than the survival patients. Furthermore, the heterogeneity was high for fatigue (I^2^=85.99%, *P*<0.1) and dyspnea (I^2^=66.89%, *P*<0.1). Thus, the random-effects model was used for the analyses. For sputum, we used the fixed-effects model because the heterogeneity was low (I^2^<50%) and the Q statistic for the test of heterogeneity indicated statistical non-significance (*P*>0.1).

The death group had a higher proportion of hemoptisis than the survival group (OR=1.40, 95% CI: 0.66-2.97, *P*=0.38). However, the former group had lower proportions of fever (OR=0.84, 95% CI: 0.57-1.21, *P*=0.34), cough (OR=0.87, 95% CI: 0.56-1.35, *P*=0.54), headache (OR=0.75, 95% CI: 0.40-1.42, *P*= 0.38), myalgiaor (OR=0.94, 95% CI: 0.73-1.23, *P*=0.68), diarrhea (OR=0.68, 95% CI: 0.39-1.19, *P*=0.18), nausea/vomiting (OR=0.83, 95% CI: 0.52-1.34, *P*=0.44), and sore throat (OR=0.61, 95% CI 0.35-1.06, *P*=0.08) than the latter group. All of these differences showed no statistical significance. With regard to myalgiaor, nausea/vomiting, and sore throat, as the heterogeneity was not significant (I^2^<50% and *P*>0.1 in the Q test), the fixed-effects model was used. However, we employed the random-effects model for the analyses of fever, cough, headache, and diarrhea because the heterogeneity was significant (I^2^≥50% or *P*<0.1 in the Q test) (Table 2 and Supplementary Figure 1).

### Comorbidities

We conducted a systematic analysis of the difference in the prevalence of comorbidities between death and survival cases. The heterogeneity test showed high heterogeneity of diabetes, hypertension, cardiovascular disease, cerebrovascular disease, and chronic renal disease, and the random-effects model was used for the meta-analysis. However, we used the fixed-effects model for malignancies, chronic obstructive pulmonary disease (COPD), and chronic liver disease because the heterogeneity levels were not significant (I^2^<50% and *P*>0.1 in the Q test) (Table 2). The death group had significantly higher proportions of patients with diabetes (OR=2.51, 95% CI: 1.86-3.35, *P*<0.001), hypertension (OR=2.39, 95% CI: 1.95-2.89, *P*<0.001), cardiovascular disease (OR=2.92, 95% CI: 2.08-4.10, *P*<0.001), chronic renal disease (OR=3.03, 95% CI: 1.77-5.16, *P*<0.001), malignancies (OR=2.36, 95% CI: 1.68-3.29, *P*<0.001), COPD (OR=1.99, 95% CI: 1.51-2.64, *P*<0.001), cerebrovascular disease (OR=2.69, 95% CI: 2.01-3.60, *P*<0.001), and chronic liver disease (OR=2.14, 95% CI: 1.60-2.86, *P*<0.001) than the survival group (Table 2 and Supplementary Figure 1).

### Laboratory examinations

The heterogeneity levels were high in the analyses of hemoglobin, platelet count, D-dimer, prothrombin time (PT), activated partial thromboplastin time (APTT), C-reactive protein (CRP), procalcitonin, and interleukin 6 (IL6) (Table 3). The results showed that death cases displayed a statistically significant lower platelet count than survival cases (MD=-35.3, 95% CI: [-58.11, -12.50], *P*<0.001). Moreover, the pooled results of these studies revealed that the death group had significantly higher levels of D-dimer (MD=5.39 μg/mL, 95% CI: 1.32, 9.46; *P*=0.01), prothrombin time (MD=1.32 s, 95% CI: 0.42-2.21, *P*<0.001), CRP (MD=63.81 mg/L, 95% CI: 38.14-89.49, *P*<0.001), procalcitonin (MD=0.64 ng/mL, 95% CI: 0.22-1.06, *P*<0.001), and interleukin 6 (MD=171.45 pg/mL, 95% CI: 66.37-276.53, *P*<0.001) than the survival group. However, the former group had higher APTT than the latter group (MD=1.19 s, 95% CI: [-0.34, 2.72], *P*=0.13), and the differences in the APTT and hemoglobin levels between the two groups were not statistically significant (Table 3 and Supplementary Figure 1).

### Imaging features

The analysis of imaging examinations is shown in Table 2. Consolidation was more likely to occur in the death group (OR = 1.80, 95% CI: 1.39-2.34, *P*<0.001). The fixed effect-model for the Ground Glass Opacity (I^2^ = 44.01%, *P*=0.13) showed that the consolidation was lower in the death group (OR =0.54, 95% CI: 0.43-0.69, *P*<0.001) (Table 2 and Supplementary Figure 1).

### Complications and supportive treatment

The random-effects model was used for acute cardiac injury, acute kidney injury, Acute Respiratory Distress Syndrome (ARDS), noninvasive ventilation and invasive mechanical ventilation because the heterogeneity was significant (I^2^≥50% or *P*<0.1 in the Q test). Meanwhile, we used the fixed-effects model for extracorporeal membrane oxygenation (ECMO) as no heterogeneity was observed (I^2^=0%, *P*=0.65). The meta-analysis showed that the death group had significantly higher proportions of patients with acute cardiac injury (OR=16.44, 95% CI: 6.23-43.82, *P*<0.001), ARDS (OR=70.81, 95% CI: 23.81-210.61, *P*<0.001), and acute kidney injury (OR=24.53, 95% CI: 4.85-125.21, *P*<0.001) than the survival group. All of the supportive treatment options were more frequently used in the death group than in the survival group (ECMO: OR=28.50, 95% CI: 5.93-135.64, *P*<0.001; noninvasive ventilation: OR =11.25, 95% CI: 1.80-70.11, *P*=0.01; and invasive mechanical ventilation: OR =24.29, 95% CI: 5.53-106.70, *P*<0.001) (Table 2 and Supplementary Figure 1).

### Subgroup analysis

Some meta-analysis results have high levels of heterogeneity. To further verify the correlation between clinical indicators and the prognosis of COVID-19 patients, subgroup analysis was performed by region. China is the country where the first outbreak occurred while some other countries reported cases later. The results of the subgroup analysis are presented in Tables 2, 3.

### Publication bias

**Figure 2:**
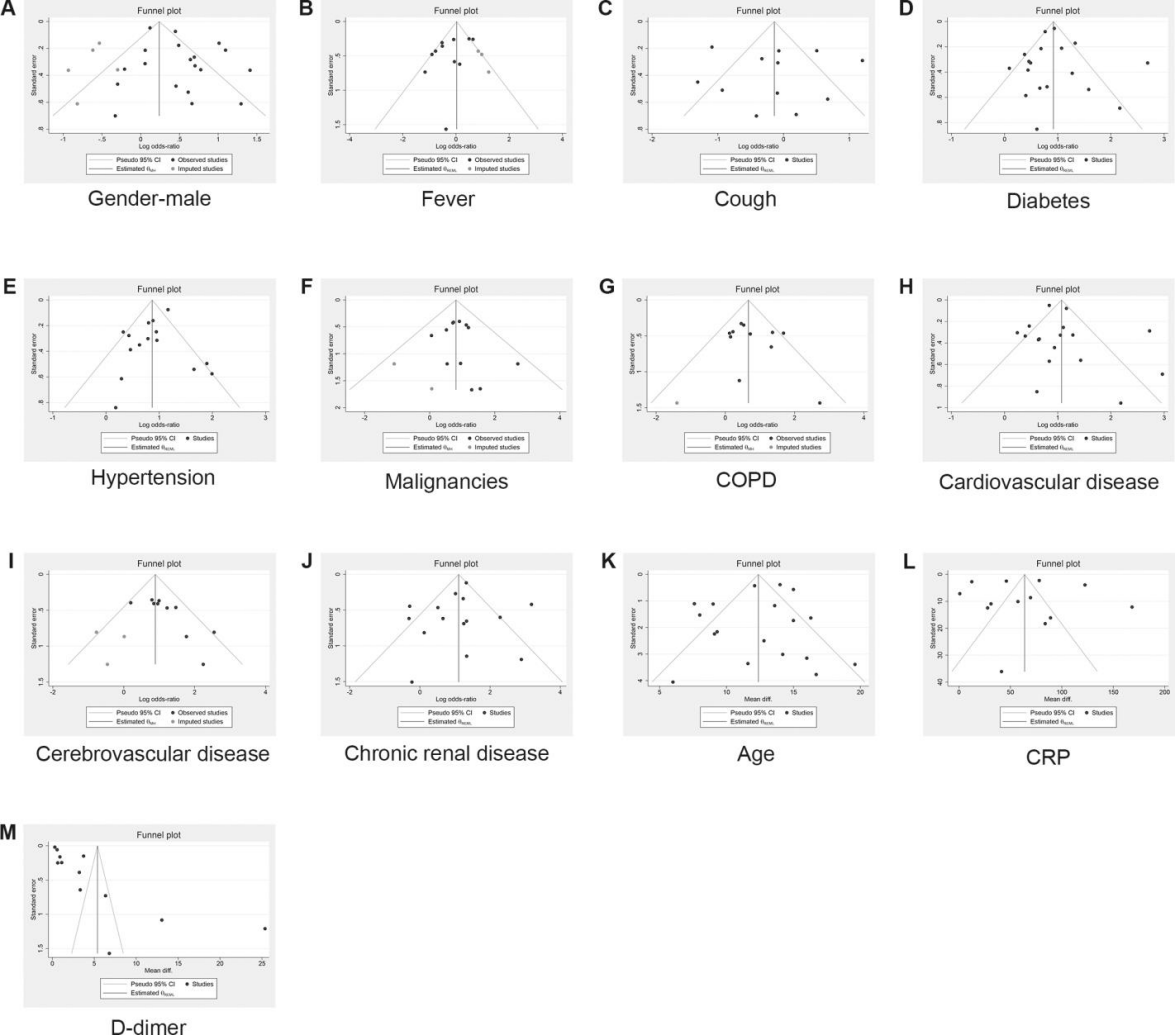
**Results of publication bias and trim and fill method.** (**A**) Gender-male; (**B**) Fever; (**C**) Cough; (**D**) Diabetes; (E) Hypertension; (**F**) Malignancies; (**G**) COPD; (**H**) Cardiovascular disease; (**I**) Cerebrovascular disease; (**J**) Chronic renal disease; (**K**) Age; (**L**) CRP; (**M**) D-dimer.

According to the number of studies (n≥10), the risk of publication bias was analyzed in the following variables: gender, age, fever, cough, diabetes, hypertension, COPD, malignancies, cardiovascular disease, cerebrovascular disease, chronic renal disease, D-dimer, and CRP. The publication bias in meta-analyses of continuous (or non-binary) variables was evaluated using the Egger’s regression test while the Harbord test was used for binary variables. All *P*-values of >0.05 suggest no significant publication bias. In addition, we addressed the potential publication bias by the trim-and-fill method. The OR values and 95% CIs before and after the application of the method are shown in Tables 4 and 5, and the funnel plots for eliminating the publication offset are presented in Figure 2.

**Table 4:** Results of trim and fill method and publication bias (binary variable).

**Variable**	**No. of Studies**	**OR(95%CI)**	**Observed + Imputed**	**P-Value**	**Method**
			**OR(95%CI)**		
Fever	11	0.84(0.57,1.21)	1.03(0.71,1.49)	0.18	Harbord
Cough	12	0.87(0.56,1.35)	0.87(0.56,1.35)	0.90	Harbord
Diabetes	18	2.51(1.86,3.35)	2.51(1.86,3.35)	0.35	Harbord
Hypertension	15	2.39(1.95,2.89)	2.39(1.95,2.89)	0.32	Harbord
Malignancies	12	2.36(1.68,3.29)	2.24(1.61,3.12)	0.85	Harbord
COPD	11	1.99(1.51,2.64)	1.95(1.47,2.58)	0.18	Harbord
Cardiovascular disease	17	2.92(2.08,4.10)	2.92(2.08,4.10)	0.29	Harbord
Cerebrovascular disease	10	2.69(2.01,3.60)	2.44(1.85,3.21)	0.01	Harbord
Chronic renal disease	15	3.03(1.77,5.16)	3.03(1.77,5.16)	0.83	Harbord

**Table 5:** Results of trim and fill method and publication bias (continuous variable).

**Variable**	**No. of Studies**	**Mean Diff.(95%CI)**	**Observed + Imputed**	**P-Value**	**Method**
			**Mean Diff.(95%CI)**		
Age	18	12.38 (10.82,13.95)	12.38 (10.82,13.95)	0.70	Egger
D-dimer	12	5.39(1.32,9.46)	5.39(1.32,9.46)	0.00	Egger
CRP	13	63.81(38.14,89.49)	63.81(38.14,89.49)	0.86	Egger

## DISCUSSION

Since December 2019, SARS-CoV-2 infection has been spreading worldwide, and a large number of confirmed cases have died from COVID-19. The current rapid spread of this infection is an emergency that primarily threatens the preparedness and biosecurity conditions of all affected countries [30]. According to previous research analyzing the cause of death of COVID-19-infected patients in Wuhan University Zhong Nan Hospital, 58.8%, 23.5%, 11.8%, and 5.9% of patients died owing to multiple organ dysfunction syndrome, cardiac arrest, respiratory failure, and acute respiratory distress syndrome, respectively [11]. The severity and outcome of COVID-19 largely depend on a patient’s age. Viable hypotheses are emerging that also include changes to the immune cell repertoire, epigenome, NAD+ levels, inflammasome activity, biological clocks, and covalent modifications of human and viral proteins [31]. Unfortunately, the knowledge regarding SARS-CoV-2 remains limited. Antiviral therapies and vaccinations are currently under development, and their effectiveness is under evaluation [32]. As the mortality rate of COVID-19 is increasing rapidly, it is of great importance to determine the general condition of infected patients and reduce the mortality rate by taking advantage of existing treatment options.

Our meta-analysis of 19 studies provides a comprehensive comparison of clinical symptoms, comorbidities, laboratory findings, chest imaging findings, complications, and supportive treatment between mortal patients and survivors. This study is expected to assist clinicians in identifying some alarming clinical characteristics presented by patients at an early stage to prevent mortality and provide instructions on the appropriate and effective management of future infections.

Many patients have been indicated to undergo nucleic acid tests on the onset of COVID-19-related symptoms. The period between the onset of symptoms and death ranged from 6 to 41 days, with a median of 14 days [33]. The most common COVID-19 symptoms at infection onset include fever, cough, and fatigue, while other symptoms are sore throat, sputum, headache, myalgia, anorexia, hemoptysis, diarrhea, and dyspnea [4, 33–35]. Notably, the transmission of SARS-COV-2 occurs during the prodromal period when infected individuals are mildly ill and undertake routine activities, contributing to the spread of infection. This indicated that a large number of asymptomatic carriers remain undiscovered [36]. It is crucial to identify and isolate asymptomatic carriers to contain outbreaks in later stages. At the same time, a prognosis should be made as soon as people present with mild symptoms to choose appropriate treatment options. Our data analysis showed that sputum, fatigue, and dyspnea occurred more significantly in mortal cases. However, the difference was not significant with other symptoms, such as fever, cough, headache, myalgia, diarrhea, hemoptysis, nausea/vomiting, and sore throat. Because of low specificity of clinical symptoms and the presence of various systemic symptoms, determining prognosis based on initial symptoms is not recommended.

According to previous studies, circulatory and endocrine comorbidities were commonly found in COVID-19 patients. Having at least one comorbidity was associated with a poor clinical outcome. Comorbidities such as chronic obstructive pulmonary disease (COPD), diabetes, hypertension, and malignancy predisposed COVID-19 patients to adverse clinical outcomes, similar to those infected with SARS-CoV and MERS-CoV, which caused severe acute respiratory syndromes. [37–41]. It is worth noting that some comorbidities frequently co-exist with each other. For instance, diabetes [42] and COPD [43] are often comorbid with hypertension or coronary heart diseases. Patients with co-existing comorbidities are more likely to have worse baseline health status; hence, it is necessary to comprehensively consider the prognostic impact of COVID-19 in patients with comorbidities. In our meta-analysis, we identified an increased risk of mortality in patients with confirmed COVID-19 who also had hypertension, diabetes, COPD, malignancies, cardiovascular disease, chronic renal disease, chronic liver disease, or cerebrovascular disease. Cardiovascular and cerebrovascular diseases were more likely to occur in middle-aged and older adults, and the mean age of the patients in the 19 included studies was more than 46 years. The physical health status of those with these comorbidities was relatively poor, thereby affecting prognosis. Furthermore, studies have indicated that ACE2 gene expression could explain why cardiovascular disease- and hypertension-predisposed patients develop a more aggressive form of COVID-19, and it is related to age, which is consistent with our analysis [44]. Patients with COPD had poor prognosis of COVID-19 because SARS-CoV-2 infection inevitably aggravated existing symptoms. In patients with chronic liver diseases, kidney diseases, or malignancies, homeostatic imbalance would further impair the functions of multiple systems after SARS-CoV-2 infection. To analyze results with high heterogeneity, we conducted country-specific subgroup analysis. This analysis supported that comorbidities such as hypertension, diabetes, COPD, malignancies, cardiovascular disease, chronic renal disease, chronic liver disease, and cerebrovascular disease were risk factors for COVID-19-related mortality. Therefore, both the category and number of comorbidities should be considered when determining the prognosis in patients with COVID-19.

Previous studies have reported laboratory examination abnormalities such as reduced lymphocyte count and elevated C-reactive protein and lactate dehydrogenase levels in patients with COVID-19 [45–47]. However, physicians cannot rely on these non-specific laboratory markers to exclude or confirm the diagnosis of COVID-19. Instead, the prognosis can be predicted using laboratory indicators. Our present study focused on coagulation as an indicator of infected patients. The results showed that platelet count in the mortal group was significantly lower than that in the survival group, and coagulation indicators, i.e., D-dimer and PT, in the former group were significantly higher than that in the latter group. This suggested that COVID-19 patients are more likely to develop coagulation disorders, for example, disseminated intravascular coagulation (DIC). In particular, the increase in the fibrin degradation product D-dimer, released from blood clots in the microvasculature, is a prognostic marker for DIC [48]. Furthermore, the relevance of thrombocytopenia in patients with DIC is associated with the risk of bleeding. Previous studies showed that 50%–60% of DIC patients had a platelet count of <100×10^9^/L, whereas 10%–15% of patients had a platelet count of <50×10^9^/L. In addition, the level of coagulation factors appears to correlate well with the severity of DIC. The low level of coagulation factors is reflected by prolonged coagulation screening tests, such as PT or APTT. A prolonged PT or APTT occurs in 14%–28% of intensive care patients but is present in more than 95% of patients with DIC [49–52]. Moreover, viral infections could cause inflammation in the body, and the increased inflammatory factors would lead to systemic immune damage and ultimately organ failure. Furthermore, hyperactivation of the immune system could cause a “cytokine storm,” which exacerbates dyspnea and hypoxemia and triggers inflammation in major tissues such as the lungs, kidneys, heart, liver, and brain. [53]. Therefore, the early detection of inflammatory indicators is integral, which was also verified in our analysis. Inflammatory indicators (i.e., CRP, procalcitonin, and IL6) in the mortal group were significantly higher than those in the survival group, showing that they can be used as prognostic clinical indicators. Our meta-analysis was limited owing to regional variation across the included studies; nevertheless, we, to a certain extent, minimized this bias through subgroup analysis.

In addition to laboratory indicators, imaging features are helpful for diagnosis and prognosis. Imaging features of viral infections usually appear as multifocal ground-glass opacities. According to a previous study, ground-glass opacities on CT corresponded to pathological diffuse alveolar damage [52]. Another study indicated that common CT imaging features in patients with COVID-19 pneumonia were bilateral and had multifocal ground-glass opacities with peripheral distribution [54]. In our meta-analysis, we found that consolidation was closely related to mortality.

According to our results, organ failure is more likely to occur in patients with poor prognosis. Therefore, monitoring the cardiopulmonary function of ICU or critical patients is highly recommended. Regarding supportive treatment of COVID-19 patients, mechanical ventilation is the most widely used short-term life support technique worldwide, and it is used on a daily basis for a diverse spectrum of indications, from scheduled surgical procedures to acute organ failure [55]. Our analysis also revealed that during the COVID-19 pandemic, mechanical ventilation and extracorporeal membrane oxygenation (ECMO) play a crucial role in the treatment of critical and fatal cases. However, a ventilator may lead to subsequent infections as bacteria can invade the lungs via the tracheostomy tube. Other problems include lung damage, pneumothorax, and an inability to discontinue ventilator support [56]. With an increasing number of patients with severe symptoms, a method for rationally and safely using mechanical ventilation needs to be urgently developed.

### Study limitations

Our current study has several limitations, namely the small number of reviewed studies, and limited data availability. The main drawback of this meta-analysis is the heterogeneity of included cases. The patients in different studies might be at different stages of disease. Furthermore, differences in the duration of observation for each study, as well as differences in treatment styles and levels across regions and hospitals, might contribute to heterogeneity. Considering the current global epidemic of COVID-19, future analysis should include more international publications. Our research will be updated when new evidence emerges.

## MATERIALS AND METHODS

### Search strategy

Our current meta-analysis was reported in accordance with the Preferred Reporting Items for Systematic Reviews and Meta-Analyses (PRISMA) Statement. We selected relevant studies published between Jan 1, 2020 and Aug 10, 2020, by searching them in PubMed, Embase, and CNKI, regardless of language of publication. The terms for the literature search were combinations of “severe acute respiratory syndrome coronavirus 2”, “SARS-CoV-2,2019 novel coronavirus”, “COVID-19”, “2019-nCoV”, “Novel coronavirus 2019”, with “fatality”, “death”, “non-survivor”, “mortality”, “fetal”, and “dead”. In conformity with the quality standards for reporting systematic reviews and meta-analyses of observational studies, two independent researchers (Zhou XH and Cheng ZP) screened retrieved articles. The researchers independently assessed full texts of articles deemed eligible for inclusion. All disagreements were resolved by discussion with a third reviewer (Hu Yu).

### Selection criteria

The inclusion criteria were as follows: (1) patients should be confirmed to have been infected with SARS-CoV-2; (2) each study should consist of a death group; (3) the full text of each article should be available; and (4) at least one outcome was reported among demographical characteristics, comorbidities, clinical characteristics, laboratory examinations, or image examinations. Meanwhile, duplicate reports, clinical guidelines, consensus documents, reviews, and systematic reviews were excluded from our meta-analysis.

### Data analysis

Two authors (Zhou XH and Cheng ZP) independently extracted relevant information, including first author, published journal, publication time, country, the number of COVID-19 patients, the mean or median age of patients, gender ratio, the presence of clinical symptoms, comorbidities, laboratory findings, image examinations, complications, and supportive treatment. All data analyses were performed using STATA 16.0 software. The ORs, mean differences, and relevant 95% CIs were used to estimate pooled results from studies. In case of no obvious heterogeneity (I^2^<50% and *P*>0.1 in the Q test), the fixed-effects model was applied. Otherwise, the random-effects model was used. All *P*-values of ≤0.05 were considered to be significant statistically. When the analysis included no less than ten articles, we performed the Egger’s regression test and the Harbord test to analyze the publication bias. Furthermore, we used the trim-and-fill method to eliminate the impact of the publication bias.

## Supplementary Material

Supplementary Figure 1

Supplementary File 1
