# Supplementary figures and images for: Characteristics of mortal COVID-19 cases compared to the survivors
Source: Aging (Albany NY). 2020 Nov 21;12(24):24579–95. doi: 10.18632/aging.202216 (PMC7803528; doi:10.18632/aging.202216)

# SUPPLEMENTARY FIGURE

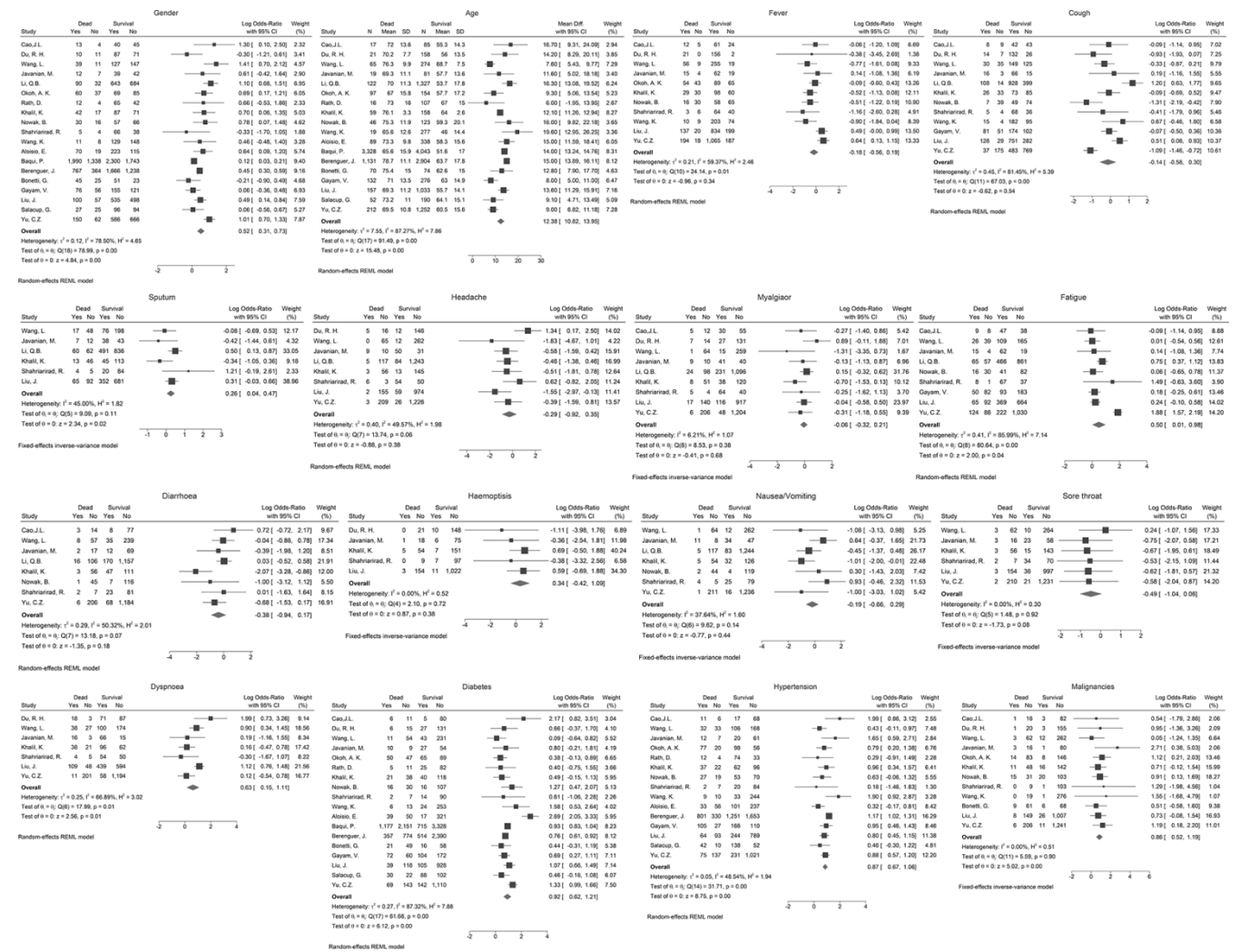

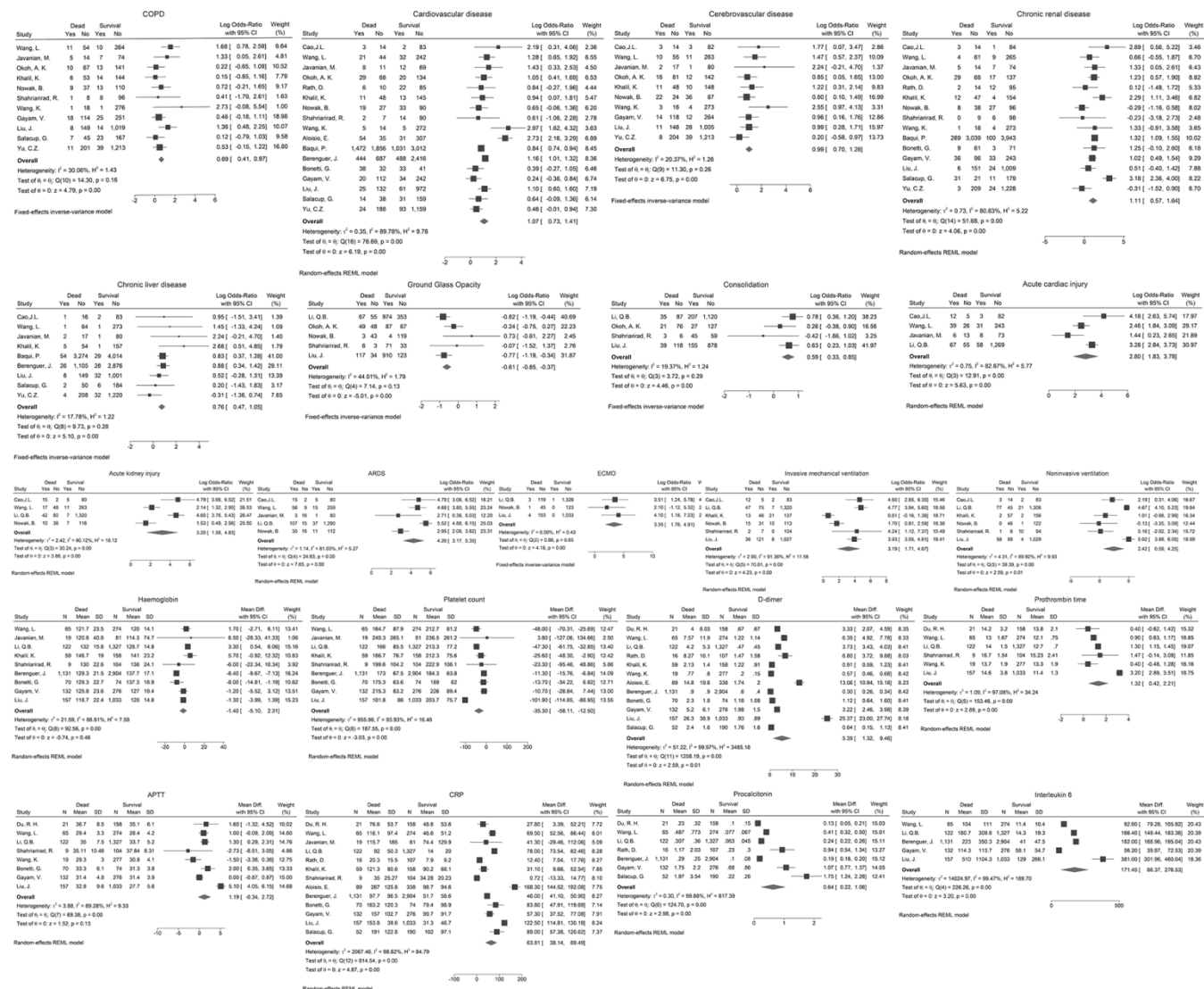

Supplement: Supplementary Figure 1 [file aging-12-202216-s001.pdf]
