# Supplementary material for: Characteristics of mortal COVID-19 cases compared to the survivors
Source: Aging (Albany NY). 2020 Nov 21;12(24):24579–95. doi: 10.18632/aging.202216 (PMC7803528; doi:10.18632/aging.202216)
Supplement: Supplementary File 1 [file aging-12-202216-s002.pdf]

Supplementary File 1. Excluded literatures in the full-text screening step.

Groups were based only on severity [1–43]

Epidemiological studies [44–55]

Only described children and youth [56–72]

Diagnosis and drug therapy [73–87]

Only described pregnant patients [88–101]

Reviews [102–113]

1. Bao C, Tao X, Cui W, Yi B, Pan T, Young KH, Qian W. SARS-CoV-2 induced thrombocytopenia as an important biomarker significantly correlated with abnormal coagulation function, increased intravascular blood clot risk and mortality in COVID-19 patients. Exp Hematol Oncol. 2020; 9:16. https://doi.org/10.1186/s40164-020-00172-4 PMID:32695551
2. Dang JZ, Zhu GY, Yang YJ, Zheng F. Clinical characteristics of coronavirus disease 2019 in patients aged 80 years and older. J Integr Med. 2020; 18:395–400. https://doi.org/10.1016/j.joim.2020.07.002 PMID:32690444
3. Hundt MA, Deng Y, Ciarleglio MM, Nathanson MH, Lim JK. Abnormal Liver Tests in COVID-19: A Retrospective Observational Cohort Study of 1827 Patients in a Major U.S. Hospital Network. Hepatology. 2020; 72:1169–76. https://doi.org/10.1002/hep.31487 PMID:32725890
4. Okada P, Buathong R, Phuygun S, Thanadachakul T, Parnmen S, Wongboot W, Waicharoen S, Wacharapluesadee S, Uttayamakul S, Vachiraphan A, Chittaganpitch M, Mekha N, Janejai N, et al. Early transmission patterns of coronavirus disease 2019 (COVID-19) in travellers from Wuhan to Thailand, January 2020. Euro Surveill. 2020; 25. https://doi.org/10.2807/1560-7917.ES.2020.25.8.2000097 PMID:32127124
5. Liao D, Zhou F, Luo L, Xu M, Wang H, Xia J, Gao Y, Cai L, Wang Z, Yin P, Wang Y, Tang L, Deng J, et al. Haematological characteristics and risk factors in the classification and prognosis evaluation of COVID-19: a retrospective cohort study. Lancet Haematol. 2020; 7:e671–78. https://doi.org/10.1016/S2352-3026(20)30217-9 PMID:32659214
6. Nie Y, Li J, Huang X, Guo W, Zhang X, Ma Y, Wang H, Qi M, Tang X, Shen X, Dai X. Epidemiological and clinical characteristics of 671 COVID-19 patients in Henan Province, China. Int J Epidemiol. 2020; 49:1085–95. https://doi.org/10.1093/ije/dyaa081 PMID:32588051
7. Scarfò L, Chatzikonstantinou T, Rigolin GM, Quaresmini G, Motta M, Vitale C, Garcia-Marco JA, Hernández-Rivas JÁ, Mirás F, Baile M, Marquet J, Niemann CU, Reda G, et al. COVID-19 severity and mortality in patients with chronic lymphocytic leukemia: a joint study by ERIC, the European Research Initiative on CLL, and CLL Campus. Leukemia. 2020; 34:2354–63. https://doi.org/10.1038/s41375-020-0959-x PMID:32647324
8. Sun Y, Dong Y, Wang L, Xie H, Li B, Chang C, Wang FS. Characteristics and prognostic factors of disease severity in patients with COVID-19: the Beijing experience. J Autoimmun. 2020; 112:102473. https://doi.org/10.1016/j.jaut.2020.102473 PMID:32439209
9. Wang B, Wang Z, Zhao J, Zeng X, Wu M, Wang S, Wang T. Epidemiological and clinical course of 483 patients with COVID-19 in Wuhan, China: a single-center, retrospective study from the mobile cabin hospital. Eur J Clin Microbiol Infect Dis. 2020; 39:2309–15. https://doi.org/10.1007/s10096-020-03927-3 PMID:32683596
10. Wu Y, Huang X, Sun J, Xie T, Lei Y, Muhammad J, Li X, Zeng X, Zhou F, Qin H, Shao L, Zhang Q. Clinical Characteristics and Immune Injury Mechanisms in 71 Patients with COVID-19. MSphere. 2020; 5:e00362-20. https://doi.org/10.1128/mSphere.00362-20 PMID:32669467
11. Zhang SY, Lian JS, Hu JH, Zhang XL, Lu YF, Cai H, Gu JQ, Ye CY, Jin CL, Yu GD, Jia HY, Zhang YM, Sheng JF, et al. Clinical characteristics of different subtypes and risk factors for the severity of illness in patients with COVID-19 in Zhejiang, China. Infect Dis Poverty. 2020; 9:85. https://doi.org/10.1186/s40249-020-00710-6 PMID:32641121
12. Cai Q, Huang D, Ou P, Yu H, Zhu Z, Xia Z, Su Y, Ma Z, Zhang Y, Li Z, He Q, Liu L, Fu Y, Chen J, et al. COVID-19 in a Designated Infectious Diseases Hospital Outside Hubei Province, China. Allergy. 2020; 75:1742-1752. https://doi.org/10.1111/all.14309 PMID:32239761
13. Chen G, Wu D, Guo W, Cao Y, Huang D, Wang H, Wang T, Zhang X, Chen H, Yu H, Zhang X, Zhang M, Wu S, et al. Clinical and immunologic features in severe and

moderate Coronavirus Disease 2019. J Clin Invest. 2020; 130:2620–29. https://doi.org/10.1172/JCI137244

14. Chen L, Liu HG, Liu W, Liu J, Liu K, Shang J, Deng Y, Wei S. [Analysis of clinical features of 29 patients with 2019 novel coronavirus pneumonia]. Zhonghua Jie He He Hu Xi Za Zhi. 2020; 43:E005. Analysis of clinical features of 29 patients with 2019 novel coronavirus pneumonia PMID:32026671
15. Chu J, Yang N, Wei Y, Yue H, Zhang F, Zhao J, He L, Sheng G, Chen P, Li G, Wu S, Zhang B, Zhang S, et al. Clinical characteristics of 54 medical staff with COVID-19: A retrospective study in a single center in Wuhan, China. J Med Virol. 2020; 92:807–13. https://doi.org/10.1002/jmv.25793 PMID:32222986
16. Gao Y, Li T, Han M, Li X, Wu D, Xu Y, Zhu Y, Liu Y, Wang X, Wang L. Diagnostic utility of clinical laboratory data determinations for patients with the severe COVID-19. J Med Virol. 2020; 92:791–96. https://doi.org/10.1002/jmv.25770 PMID:32181911
17. Guan WJ, Ni ZY, Hu Y, Liang WH, Ou CQ, He JX, Liu L, Shan H, Lei CL, Hui DS, Du B, Li LJ, Zeng G, et al. Clinical Characteristics of Coronavirus Disease 2019 in China. N Engl J Med. 2020; 382:1708-1720. https://doi.org/10.1056/NEJMoa2002032 PMID:32109013
18. Han H, Xie L, Liu R, Yang J, Liu F, Wu K, Chen L, Hou W, Feng Y, Zhu C. Analysis of heart injury laboratory parameters in 273 COVID-19 patients in one hospital in Wuhan, China. J Med Virol. 2020; 92:819–23. https://doi.org/10.1002/jmv.25809 PMID:32232979
19. Ji D, Zhang D, Xu J, Chen Z, Yang T, Zhao P, Chen G, Cheng G, Wang Y, Bi J, Tan L, Lau G, Qin E. Prediction for Progression Risk in Patients With COVID-19 Pneumonia: the CALL Score. Clin Infect Dis. 2020; 71:1393–99. https://doi.org/10.1093/cid/ciaa414 PMID:32271369
20. Li K, Fang Y, Li W, Pan C, Qin P, Zhong Y, Liu X, Huang M, Liao Y, Li S. CT image visual quantitative evaluation and clinical classification of coronavirus disease (COVID-19). Eur Radiol. 2020; 30:4407–16. https://doi.org/10.1007/s00330-020-06817-6 PMID:32215691
21. Li K, Wu J, Wu F, Guo D, Chen L, Fang Z, Li C. The Clinical and Chest CT Features Associated With Severe and Critical COVID-19 Pneumonia. Invest Radiol. 2020; 55:327–31. https://doi.org/10.1097/RLI.0000000000000672 PMID:32118615
22. Liu C, Jiang ZC, Shao CX, Zhang HG, Yue HM, Chen ZH, Ma BY, Liu WY, Huang HH, Yang J, Wang Y, Liu HY, Xu D, et al. [Preliminary study of the relationship between novel coronavirus pneumonia and liver function damage: a multicenter study.] Zhonghua Gan Zang Bing Za Zhi. 2020; 28:148–52. https://doi.org/10.3760/cma.j.issn.1007-3418.2020.02.003 PMID:32077660
23. Liu KC, Xu P, Lv WF, Qiu XH, Yao JL, Gu JF, Wei W. CT manifestations of coronavirus disease-2019: A retrospective analysis of 73 cases by disease severity. Eur J Radiol. 2020; 126:108941. https://doi.org/10.1016/j.ejrad.2020.108941 PMID:32193037
24. Liu M, He P, Liu HG, Wang XJ, Li FJ, Chen S, Lin J, Chen P, Liu JH, Li CH. [Clinical characteristics of 30 medical workers infected with new coronavirus pneumonia]. Zhonghua Jie He He Hu Xi Za Zhi. 2020; 43:209–14. Clinical characteristics of 30 medical workers infected with new coronavirus pneumonia PMID:32164090
25. Liu W, Tao ZW, Wang L, Yuan ML, Liu K, Zhou L, Wei S, Deng Y, Liu J, Liu HG, Yang M, Hu Y. Analysis of factors associated with disease outcomes in hospitalized patients with 2019 novel coronavirus disease. Chin Med J (Engl), 2020; 133:1032-1038. https://doi.org/10.1097/CM9.0000000000000775. PMID:32118640
26. Lo IL, Lio CF, Cheong HH, Lei CI, Cheong TH, Zhong X, Tian Y, Sin NN. Evaluation of SARS-CoV-2 RNA shedding in clinical specimens and clinical characteristics of 10 patients with COVID-19 in Macau. Int J Biol Sci. 2020; 16:1698–707. https://doi.org/10.7150/ijbs.45357 PMID:32226287
27. Mo P, Xing Y, Xiao Y, Deng L, Zhao Q, Wang H, Xiong Y, Cheng Z, Gao S, Liang K, Luo M, Chen T, Song S, et al., Clinical characteristics of refractory COVID-19 pneumonia in Wuhan, China. Clin Infect Dis, 2020. [Epub ahead of print]. https://doi.org/10.1093/cid/ciaa270 PMID:32173725
28. Peng YD, Meng K, Guan HQ, Leng L, Zhu RR, Wang BY, He MA, Cheng LX, Huang K, Zeng QT. [Clinical characteristics and outcomes of 112 cardiovascular disease patients infected by 2019-nCoV.] Zhonghua Xin Xue Guan Bing Za Zhi. 2020; 48:E004. https://doi.org/10.3760/cma.j.cn112148-20200220-00105 PMID:32120458
29. Qian ZP, Mei X, Zhang YY, Zou Y, Zhang ZG, Zhu H, Guo HY, Liu Y, Ling Y, Zhang XY, Wang JF, Lu HZ. [Analysis of baseline liver biochemical parameters in 324 cases with novel coronavirus pneumonia in Shanghai area]. Zhonghua Gan Zang Bing Za Zhi. 2020; 28:E005. https://doi.org/10.3760/cma.j.cn501113-20200229-00076 PMID:32270660
30. Qin C, Zhou L, Hu Z, Zhang S, Yang S, Tao Y, Xie C, Ma K, Shang K, Wang W, Tian DS. Dysregulation of immune response in patients with COVID-19 in Wuhan, China. Clin Infect Dis. 2020; 71:762-768.

https://doi.org/10.1093/cid/ciaa248 PMID:32161940

31. Qu R, Ling Y, Zhang YH, Wei LY, Chen X, Li XM, Liu XY, Liu HM, Guo Z, Ren H, Wang Q. Platelet-to-lymphocyte ratio is associated with prognosis in patients with coronavirus disease-19. J Med Virol. 2020; 92:1533–41. https://doi.org/10.1002/jmv.25767 PMID:32181903

32. Tian S, Hu N, Lou J, Chen K, Kang X, Xiang Z, Chen H, Wang D, Liu N, Liu D, Chen G, Zhang Y, Li D, et al. Characteristics of COVID-19 infection in Beijing. J Infect. 2020; 80:401–06. https://doi.org/10.1016/j.jinf.2020.02.018 PMID:32112886

33. Wan S, Xiang Y, Fang W, Zheng Y, Li B, Hu Y, Lang C, Huang D, Sun Q, Xiong Y, Huang X, Lv J, Luo Y, et al. Clinical features and treatment of COVID-19 patients in northeast Chongqing. J Med Virol. 2020; 92:797–806. https://doi.org/10.1002/jmv.25783 PMID:32198776

34. Wang D, Hu B, Hu C, Zhu F, Liu X, Zhang J, Wang B, Xiang H, Cheng Z, Xiong Y, Zhao Y, Li Y, Wang X, et al. Clinical Characteristics of 138 Hospitalized Patients With 2019 Novel Coronavirus-Infected Pneumonia in Wuhan, China. JAMA. 2020; 323:1061-1069. https://doi.org/10.1001/jama.2020.1585 PMID:32031570

35. Wang X, Fang J, Zhu Y, Chen L, Ding F, Zhou R, Ge L, Wang F, Chen Q, Zhang Y, Zhao Q. Clinical characteristics of non-critically ill patients with novel coronavirus infection (COVID-19) in a Fangcang Hospital. Clin Microbiol Infect. 2020; 26:1063–68. https://doi.org/10.1016/j.cmi.2020.03.032 PMID:32251842

36. Wu J, Li W, Shi X, Chen Z, Jiang B, Liu J, Wang D, Liu C, Meng Y, Cui L, Yu J, Cao H, Li L. Early antiviral treatment contributes to alleviate the severity and improve the prognosis of patients with novel coronavirus disease (COVID-19). J Intern Med. 2020; 288:128–38. https://doi.org/10.1111/joim.13063 PMID:32220033

37. Xie H, Zhao J, Lian N, Lin S, Xie Q, Zhuo H. Clinical characteristics of non-ICU hospitalized patients with coronavirus disease 2019 and liver injury: A retrospective study. Liver Int. 2020; 40:1321–26. https://doi.org/10.1111/liv.14449 PMID:32239591

38. Xu YH, Dong JH, An WM, Lv XY, Yin XP, Zhang JZ, Dong L, Ma X, Zhang HJ, Gao BL. Clinical and computed tomographic imaging features of novel coronavirus pneumonia caused by SARS-CoV-2. J Infect. 2020; 80:394–400. https://doi.org/10.1016/j.jinf.2020.02.017 PMID:32109443

39. Yuan J, Zou R, Zeng L, Kou S, Lan J, Li X, Liang Y, Ding X, Tan G, Tang S, Liu L, Liu Y, Pan Y, Wang Z. The correlation between viral clearance and biochemical outcomes of 94 COVID-19 infected discharged patients. Inflamm Res. 2020; 69:599–606. https://doi.org/10.1007/s00011-020-01342-0 PMID:32227274

40. Zhang JJ, Dong X, Cao YY, Yuan YD, Yang YB, Yan YQ, Akdis CA, Gao YD.Clinical characteristics of 140 patients infected with SARS-CoV-2 in Wuhan, China. Allergy. 2020; 75:1730-1741. https://doi.org/10.1111/all.14238 PMID:32077115

41. Zhang Y, Zheng L, Liu L, Zhao M, Xiao J, Zhao Q. Liver impairment in COVID-19 patients: a retrospective analysis of 115 cases from a single center in Wuhan city, China. Liver Int. 2020; 40:2095-2103. https://doi.org/10.1111/liv.14455 PMID:32239796

42. Zhao J, Yuan Q, Wang H, Liu W, Liao X, Su Y, Wang X, Yuan J, Li T, Li J, Qian S, Hong C, Wang F, et al. Antibody responses to SARS-CoV-2 in patients of novel coronavirus disease 2019. Clin Infect Dis. 2020; 71:2027–34. PMID:32221519

43. Zheng C, Wang J, Guo H, Lu Z, Ma Y, Zhu Y, Xia D, Wang Y, He H, Zhou J, Wang Y, Fei M, Yin Y, et al, and Anhui Medical team members of National aid to prevent and treat novel coronavirus pneumonia in Wuhan. Risk-adapted Treatment Strategy For COVID-19 Patients. Int J Infect Dis. 2020; 94:74–77. https://doi.org/10.1016/j.ijid.2020.03.047 PMID:32229257

44. Jia J, Hu X, Yang F, Song X, Dong L, Zhang J, Jiang F, Gao R. Epidemiological characteristics on the clustering nature of COVID-19 in Qingdao City, 2020: a descriptive analysis. Disaster Med Public Health Prep. 2020; 31:1–5. https://doi.org/10.1017/dmp.2020.59 PMID:32228732

45. Li XQ, Cai WF, Huang LF, Chen C, Liu YF, Zhang ZB, Yuan J, Li TG, Wang M. [Comparison of epidemic characteristics between SARS in 2003 and COVID-19 in 2020 in Guangzhou]. Zhonghua Liu Xing Bing Xue Za Zhi. 2020; 41:634–37. Comparison of epidemic characteristics between SARS in2003 and COVID-19 in 2020 in Guangzhou PMID:32159317

46. Liang WH, Guan WJ, Li CC, Li YM, Liang HR, Zhao Y, Liu XQ, Sang L, Chen RC, Tang CL, Wang T, Wang W, He QH, et al. Clinical characteristics and outcomes of hospitalised patients with COVID-19 treated in Hubei (epicentre) and outside Hubei (non-epicentre): a nationwide analysis of China. Eur Respir J. 2020; 55:2000562. https://doi.org/10.1183/13993003.00562-2020 PMID:32269086

47. Park SE. Epidemiology, virology, and clinical features of severe acute respiratory syndrome -coronavirus-2

(SARS-CoV-2; Coronavirus Disease-19). Clin Exp Pediatr; 2020.

48. Qiu YY, Wang SQ, Wang XL, Lu WX, Qiao D, Li JB, Gu YY, Zeng Y, Chen Y, Bai WZ, Xu BL, Han TW. [Epidemiological analysis on a family cluster of COVID-19.] Zhonghua Liu Xing Bing Xue Za Zhi. 2020; 41:506–09. https://doi.org/10.3760/cma.j.cn112338-20200221-00147 PMID:32133831

49. Wang L, Gao YH, Lou LL, Zhang GJ. The clinical dynamics of 18 cases of COVID-19 outside of Wuhan, China. Eur Respir J. 2020; 55:2000398. https://doi.org/10.1183/13993003.00398-2020 PMID:32139464

50. Wu WS, Li YG, Wei ZF, Zhou PH, Lyu LK, Zhang GP, Zhao Y, He HY, Li XY, Gao L, Zhang XM, Liu H, Zhou N, et al. [Investigation and analysis on characteristics of a cluster of COVID-19 associated with exposure in a department store in Tianjin]. Zhonghua Liu Xing Bing Xue Za Zhi. 2020; 41:489–93. Investigation and analysis on characteristics of a cluster of COVID-19 associated with exposure in a department store in Tianjin PMID:32133830

51. Zheng Y, Xiong C, Liu Y, Qian X, Tang Y, Liu L, Leung EL, Wang M. Epidemiological and clinical characteristics analysis of COVID-19 in the surrounding areas of Wuhan, Hubei Province in 2020. Pharmacol Res. 2020; 157:104821. https://doi.org/10.1016/j.phrs.2020.104821 PMID:32360481

52. Giesen C, Diez-Izquierdo L, Saa-Requejo CM, Lopez-Carrillo I, Lopez-Vilela CA, Seco-Martinez A, Prieto MT, Malmierca E, Garcia-Fernandez C, and COVID Epidemiological Surveillance and Control Study Group. Epidemiological characteristics of the COVID-19 outbreak in a secondary hospital in Spain. Am J Infect Control. 2020S0196-6553(20)30700-8. https://doi.org/10.1016/j.ajic.2020.07.014 PMID:32663494

53. Khamis F, Al Rashidi B, Al-Zakwani I, Al Wahaibi AH, Al Awaidy ST. Epidemiology of COVID-19 Infection in Oman: Analysis of the First 1304 Cases. Oman Med J. 2020; 35:e145. https://doi.org/10.5001/omj.2020.60 PMID:32647593

54. Lee JY, Hong SW, Hyun M, Park JS, Lee JH, Suh YS, Kim DH, Han SW, Cho CH, Kim HA, and Epidemiological and Clinical Characteristics of Coronavirus Disease 2019 in Daegu, South Korea. Epidemiological and clinical characteristics of coronavirus disease 2019 in Daegu, South Korea. Int J Infect Dis. 2020; 98:462–66. https://doi.org/10.1016/j.ijid.2020.07.017 PMID:32702415

55. Tsou TP, Chen WC, Huang AS, Chang SC, Chen WC, Huang AS, Su CP, Tsou TP, Lee PH, Chan PC, Wu HH, Huang ST, Su WJ, et al, and Taiwan COVID-19 Outbreak Investigation Team. Epidemiology of the first 100 cases of COVID-19 in Taiwan and its implications on outbreak control. J Formos Med Assoc. 2020; 119:1601–07. https://doi.org/10.1016/j.jfma.2020.07.015 PMID:32718892

56. Feng K, Yun YX, Wang XF, Yang GD, Zheng YJ, Lin CM, Wang LF. [Analysis of CT features of 15 Children with 2019 novel coronavirus infection]. Zhonghua Er Ke Za Zhi. 2020; 58:E007. Analysis of CT features of 15 Children with 2019 novel coronavirus infection PMID:32061200

57. Qiu H, Wu J, Hong L, Luo Y, Song Q, Chen D. Clinical and epidemiological features of 36 children with coronavirus disease 2019 (COVID-19) in Zhejiang, China: an observational cohort study. Lancet Infect Dis. 2020; 20:689–96. https://doi.org/10.1016/S1473-3099(20)30198-5 PMID:32220650

58. Shen Q, Guo W, Guo T, Li J, He W, Ni S, Ouyang X, Liu J, Xie Y, Tan X, Zhou Z, Peng H. Novel coronavirus infection in children outside of Wuhan, China. Pediatr Pulmonol. 2020; 55:1424-1429. https://doi.org/10.1002/ppul.24762 PMID:32259403

59. Sun D, Li H, Lu XX, Xiao H, Ren J, Zhang FR, Liu ZS. Clinical features of severe pediatric patients with coronavirus disease 2019 in Wuhan: a single center's observational study. World J Pediatr. 2020; 16:251–59. https://doi.org/10.1007/s12519-020-00354-4 PMID:32193831

60. Wang D, Ju XL, Xie F, Lu Y, Li FY, Huang HH, Fang XL, Li YJ, Wang JY, Yi B, Yue JX, Wang J, Wang LX, et al. [Clinical analysis of 31 cases of 2019 novel coronavirus infection in children from six provinces (autonomous region) of northern China.] Zhonghua Er Ke Za Zhi. 2020; 58:269-74. https://doi.org/10.3760/cma.j.cn112140-20200225-00138 PMID:32118389

61. Wang XF, Yuan J, Zheng YJ, Chen J, Bao YM, Wang YR, Wang LF, Li H, Zeng JX, Zhang YH, Liu YX, Liu L. [Retracted: clinical and epidemiological characteristics of 34 children with 2019 novel coronavirus infection in Shenzhen]. Zhonghua Er Ke Za Zhi. 2020; 58:E008. Retracted: Clinical and epidemiological characteristics of 34 children with 2019 novel coronavirus infection in Shenzhen PMID:32062875

62. Xia W, Shao J, Guo Y, Peng X, Li Z, Hu D. Clinical and CT features in pediatric patients with COVID-19 infection: different points from adults. Pediatr Pulmonol. 2020; 55:1169–74. https://doi.org/10.1002/ppul.24718 PMID:32134205

63. Zheng F, Liao C, Fan QH, Chen HB, Zhao XG, Xie ZG, Li XL, Chen CX, Lu XX, Liu ZS, Lu W, Chen CB, Jiao R, et al. Clinical Characteristics of Children with Coronavirus Disease 2019 in Hubei, China. Curr Med Sci. 2020; 40:275-280. https://doi.org/10.1007/s11596-020-2172-6 PMID:32207032

64. Zhou Y, Yang GD, Feng K, Huang H, Yun YX, Mou XY, Wang LF. [Clinical features and chest CT findings of coronavirus disease 2019 in infants and young children]. Zhongguo Dang Dai Er Ke Za Zhi. 2020; 22:215–20. Clinical features and chest CT findings of coronavirus disease 2019 in infants and young children PMID:32204756

65. Zhu L, Wang J, Huang R, Liu L, Zhao H, Wu C, Zhu C. Clinical characteristics of a case series of children with coronavirus disease 2019. Pediatr Pulmonol. 2020; 55:1430–32. https://doi.org/10.1002/ppul.24767 PMID:32270592

66. Zhang L, Peres TG, Silva MV, Camargos P. What we know so far about Coronavirus Disease 2019 in children: A meta-analysis of 551 laboratory-confirmed cases. Pediatr Pulmonol. 2020; 55:2115–27. https://doi.org/10.1002/ppul.24869 PMID:32519809

67. Götzinger F, Santiago-García B, Noguera-Julián A, Lanaspa M, Lancella L, Calò Carducci FI, Gabrovska N, Velizarova S, Prunk P, Osterman V, Krivec U, Lo Vecchio A, Shingadia D, et al, and ptbnet COVID-19 Study Group. COVID-19 in children and adolescents in Europe: a multinational, multicentre cohort study. Lancet Child Adolesc Health. 2020; 4:653–61. https://doi.org/10.1016/S2352-4642(20)30177-2 PMID:32593339

68. Lee PY, Day-Lewis M, Henderson LA, Friedman KG, Lo J, Roberts JE, Lo MS, Platt CD, Chou J, Hoyt KJ, Baker AL, Banzon TM, Chang MH, et al. Distinct clinical and immunological features of SARS-CoV-2-induced multisystem inflammatory syndrome in children. J Clin Invest. 2020; 130:5942–50. https://doi.org/10.1172/JCI141113 PMID:32701511

69. Bialek S, Gierke R, Hughes M, McNamara LA, Pilishvili T, Skoff T, and CDC COVID-19 Response Team. Coronavirus Disease 2019 in Children - United States, February 12-April 2, 2020. MMWR Morb Mortal Wkly Rep. 2020; 69:422–26. https://doi.org/10.15585/mmwr.mm6914e4 PMID:32271728

70. Chen XB, Du SH, Lu JC, Tan XH, Li DR, Yue X, Wang Q, Wang HJ, Qiao DF. Retrospective Analysis of 61 Cases of Children Died of Viral Pneumonia. Fa Yi Xue Za Zhi. 2020; 36:164–68. PMID:32530160

71. Liu H, Liu F, Li J, Zhang T, Wang D, Lan W. Clinical and CT imaging features of the COVID-19 pneumonia: focus on pregnant women and children. J Infect. 2020; 80:e7–13. https://doi.org/10.1016/j.jinf.2020.03.007 PMID:32171865

72. Su L, Ma X, Yu H, Zhang Z, Bian P, Han Y, Sun J, Liu Y, Yang C, Geng J, Zhang Z, Gai Z. The different clinical characteristics of corona virus disease cases between children and their families in China - the character of children with COVID-19. Emerg Microbes Infect. 2020; 9:707–13. https://doi.org/10.1080/22221751.2020.1744483 PMID:32208917

73. Huang G, Gong T, Wang G, Wang J, Guo X, Cai E, Li S, Li X, Yu Y, Lin L. Timely Diagnosis and Treatment Shortens the Time to Resolution of Coronavirus Disease (COVID-19) Pneumonia and Lowers the Highest and Last CT Scores From Sequential Chest CT. AJR Am J Roentgenol. 2020; 215:367–73. https://doi.org/10.2214/AJR.20.23078 PMID:32223665

74. Li L, Qin L, Xu Z, Yin Y, Wang X, Kong B, Bai J, Lu Y, Fang Z, Song Q, Cao K, Liu D, Wang G, et al. Artificial Intelligence Distinguishes COVID-19 from Community Acquired Pneumonia on Chest CT. Radiology. 2020; 30:200905. https://doi.org/10.1148/radiol.2020200905.

75. Mardani R, Ahmadi Vasmehjani A, Zali F, Gholami A, Mousavi Nasab SD, Kaghazian H, Kaviani M, Ahmadi N, and Laboratory Parameters in Detection of COVID-19 Patients with Positive RT-PCR. Laboratory Parameters in Detection of COVID-19 Patients with Positive RT-PCR; a Diagnostic Accuracy Study. Arch Acad Emerg Med. 2020; 8:e43. PMID:32259132

76. Xiong Z, Fu L, Zhou H, Liu JK, Wang AM, Huang Y, Huang X, Yi B, Wu J, Li CH, Quan J, Li M, Leng YS, et al. [Construction and evaluation of a novel diagnosis process for 2019-Corona Virus Disease]. Zhonghua Yi Xue Za Zhi. 2020; 100:1223-1229. https://doi.org/10.3760/cma.j.cn112137-20200228-00499 PMID:32157849

77. Zhu W, Xie K, Lu H, Xu L, Zhou S, Fang S. Initial clinical features of suspected coronavirus disease 2019 in two emergency departments outside of Hubei, China. J Med Virol. 2020; 92:1525–32. https://doi.org/10.1002/jmv.25763 PMID:32167181

78. Luo P, Liu Y, Qiu L, Liu X, Liu D, Li J. Tocilizumab treatment in COVID-19: A single center experience. J Med Virol. 2020; 92:814–18. https://doi.org/10.1002/jmv.25801 PMID:32253759

79. Cao Y, Wei J, Zou L, Jiang T, Wang G, Chen L, Huang L, Meng F, Huang L, Wang N, Zhou X, Luo H, Mao Z, et al.

Ruxolitinib in treatment of severe coronavirus disease 2019 (COVID-19): A multicenter, single-blind, randomized controlled trial. J Allergy Clin Immunol. 2020; 146:137–146.e3.
https://doi.org/10.1016/j.jaci.2020.05.019
PMID:32470486

80. Lecronier M, Beurton A, Burrel S, Haudebourg L, Deleris R, Le Marec J, Virolle S, Nemlaghi S, Bureau C, Mora P, De Sarcus M, Clovet O, Duceau B, et al. Comparison of hydroxychloroquine, lopinavir/ritonavir, and standard of care in critically ill patients with SARS-CoV-2 pneumonia: an opportunistic retrospective analysis. Crit Care. 2020; 24:418.
https://doi.org/10.1186/s13054-020-03117-9
PMID:32653015

81. Olender SA, Perez KK, Go AS, Balani B, Price-Haywood EG, Shah NS, Wang S, Walunas TL, Swaminathan S, Slim J, Chin B, De Wit S, Ali SM, et al. Remdesivir for Severe COVID-19 versus a Cohort Receiving Standard of Care. Clin Infect Dis. 2020ciaa1041.
https://doi.org/10.1093/cid/ciaa1041 PMID:32706859

82. Tong S, Su Y, Yu Y, Wu C, Chen J, Wang S, Jiang J. Ribavirin therapy for severe COVID-19: a retrospective cohort study. Int J Antimicrob Agents. 2020; 56:106114.
https://doi.org/10.1016/j.ijantimicag.2020.106114
PMID:32712334

83. Yang G, Tan Z, Zhou L, Yang M, Peng L, Liu J, Cai J, Yang R, Han J, Huang Y, He S. Effects of Angiotensin II Receptor Blockers and ACE (Angiotensin-Converting Enzyme) Inhibitors on Virus Infection, Inflammatory Status, and Clinical Outcomes in Patients With COVID-19 and Hypertension: A Single-Center Retrospective Study. Hypertension. 2020; 76:51–58.
https://doi.org/10.1161/HYPERTENSIONAHA.120.15143 PMID:32348166

84. Yekedüz E, Dursun B, Aydın GÇ, Yazgan SC, Öztürk HH, Azap A, Utkan G, Ürün Y. Clinical course of COVID-19 infection in elderly patient with melanoma on nivolumab. J Oncol Pharm Pract. 2020; 26:1289–94.
https://doi.org/10.1177/1078155220924084
PMID:32423324

85. Cao B, Wang Y, Wen D, Liu W, Wang J, Fan G, Ruan L, Song B, Cai Y, Wei M, Li X, Xia J, Chen N, et al. A Trial of Lopinavir-Ritonavir in Adults Hospitalized with Severe Covid-19. N Engl J Med. 2020; 382:1787–99.
https://doi.org/10.1056/NEJMoa2001282
PMID:32187464

86. Monteil V, Kwon H, Prado P, Hagelkrüys A, Wimmer RA, Stahl M, Leopoldi A, Garreta E, Hurtado Del Pozo C, Prosper F, Romero JP, Wirnsberger G, Zhang H, et al. Inhibition of SARS-CoV-2 Infections in Engineered Human Tissues Using Clinical-Grade Soluble Human ACE2. Cell. 2020; 181:905–913.e7.
https://doi.org/10.1016/j.cell.2020.04.004
PMID:32333836

87. Xu X, Han M, Li T, Sun W, Wang D, Fu B, Zhou Y, Zheng X, Yang Y, Li X, Zhang X, Pan A, Wei H. Effective treatment of severe COVID-19 patients with tocilizumab. Proc Natl Acad Sci USA. 2020; 117:10970–75.
https://doi.org/10.1073/pnas.2005615117
PMID:32350134

88. Chen H, Guo J, Wang C, Luo F, Yu X, Zhang W, Li J, Zhao D, Xu D, Gong Q, Liao J, Yang H, Hou W, Zhang Y. Clinical characteristics and intrauterine vertical transmission potential of COVID-19 infection in nine pregnant women: a retrospective review of medical records. Lancet. 2020; 395:809–15.
https://doi.org/10.1016/S0140-6736(20)30360-3
PMID:32151335

89. Chen S, Liao E, Cao D, Gao Y, Sun G, Shao Y. Clinical analysis of pregnant women with 2019 novel coronavirus pneumonia. J Med Virol. 2020; 92:1556–61.
https://doi.org/10.1002/jmv.25789 PMID:32222119

90. Li N, Han L, Peng M, Lv Y, Ouyang Y, Liu K, Yue L, Li Q, Sun G, Chen L, Yang L. Maternal and neonatal outcomes of pregnant women with COVID-19 pneumonia: a case-control study. Clin Infect Dis. 2020; 71:2035-2041.
https://doi.org/10.1093/cid/ciaa352 PMID:32249918

91. Schwartz DA. An Analysis of 38 Pregnant Women with COVID-19, Their Newborn Infants, and Maternal-Fetal Transmission of SARS-CoV-2: Maternal Coronavirus Infections and Pregnancy Outcomes. Arch Pathol Lab Med. 2020; 144:799–805.
https://doi.org/10.5858/arpa.2020-0901-SA
PMID:32180426

92. Yu N, Li W, Kang Q, Xiong Z, Wang S, Lin X, Liu Y, Xiao J, Liu H, Deng D, Chen S, Zeng W, Feng L, Wu J. Clinical features and obstetric and neonatal outcomes of pregnant patients with COVID-19 in Wuhan, China: a retrospective, single-centre, descriptive study. Lancet Infect Dis. 2020; 20:559–64.
https://doi.org/10.1016/S1473-3099(20)30176-6
PMID:32220284

93. Berthelot N, Lemieux R, Garon-Bissonnette J, Drouin-Maziade C, Martel É, Maziade M. Uptrend in distress and psychiatric symptomatology in pregnant women during the coronavirus disease 2019 pandemic. Acta Obstet Gynecol Scand. 2020; 99:848–55.
https://doi.org/10.1111/aogs.13925
PMID:32449178

94. Fox NS, Melka S. COVID-19 in Pregnant Women: Case Series from One Large New York City Obstetrical Practice. Am J Perinatol. 2020; 37:1002–04.

https://doi.org/10.1055/s-0040-1712529 PMID:32438425

95. Yan J, Guo J, Fan C, Juan J, Yu X, Li J, Feng L, Li C, Chen H, Qiao Y, Lei D, Wang C, Xiong G, et al. Coronavirus disease 2019 in pregnant women: a report based on 116 cases. Am J Obstet Gynecol. 2020; 223:111.e1–14. https://doi.org/10.1016/j.ajog.2020.04.014 PMID:32335053

96. Chen X, Li Y, Wang J, Cai H, Cao H, Sheng J. [Pregnant women complicated with COVID-19: a clinical analysis of 3 cases]. Zhejiang Da Xue Xue Bao Yi Xue Ban. 2020; 49:240–44. Pregnant women complicated with COVID-19: a clinical analysis of 3 cases PMID:32391671

97. Fox NS, Melka S. COVID-19 in Pregnant Women: Case Series from One Large New York City Obstetrical Practice. Am J Perinatol. 2020; 37:1002–04. https://doi.org/10.1055/s-0040-1712529 PMID:32438425

98. Knight M, Bunch K, Vousden N, Morris E, Simpson N, Gale C, O'Brien P, Quigley M, Brocklehurst P, Kurinczuk JJ, and UK Obstetric Surveillance System SARS-CoV-2 Infection in Pregnancy Collaborative Group. Characteristics and outcomes of pregnant women admitted to hospital with confirmed SARS-CoV-2 infection in UK: national population based cohort study. BMJ. 2020; 369:m2107. https://doi.org/10.1136/bmj.m2107 PMID:32513659

99. Yang H, Hu B, Zhan S, Yang LY, Xiong G. Effects of SARS-CoV-2 infection on pregnant women and their infants: A retrospective study in Wuhan, China. Arch Pathol Lab Med.2020; 144:1217-1222. https://doi.org/10.5858/arpa.2020-0232-SA PMID:32422078

100. Yu N, Li W, Kang Q, Xiong Z, Wang S, Lin X, Liu Y, Xiao J, Liu H, Deng D, Chen S, Zeng W, Feng L, Wu J. Clinical features and obstetric and neonatal outcomes of pregnant patients with COVID-19 in Wuhan, China: a retrospective, single-centre, descriptive study. Lancet Infect Dis. 2020; 20:559–64. https://doi.org/10.1016/S1473-3099(20)30176-6 PMID:32220284

101. Zhang L, Jiang Y, Wei M, Cheng BH, Zhou XC, Li J, Tian JH, Dong L, Hu RH. [Analysis of the pregnancy outcomes in pregnant women with COVID-19 in Hubei Province]. Zhonghua Fu Chan Ke Za Zhi. 2020; 55:166-171. https://doi.org/10.3760/cma.j.cn112141-20200218-00111 PMID:32145714

102. Afshar ZM, Dayani M, Naderi M, Ghanbarveisi F, Shiri S, Rajati F. Fatality rate of COVID-19 in patients with malignancies: a sytematic review and meta-analysis. J Infect. 2020; 81:e114–16. https://doi.org/10.1016/j.jinf.2020.05.062 PMID:32474042

103. Aikawa T, Takagi H, Ishikawa K, Kuno T. Myocardial injury characterized by elevated cardiac troponin and in-hospital mortality of COVID-19: an insight from a meta-analysis. J Med Virol. 2020jmv.26108. https://doi.org/10.1002/jmv.26108 PMID:32484975

104. Antwi-Amoabeng D, Kanji Z, Ford B, Beutler BD, Riddle MS, Siddiqui F. Clinical outcomes in COVID-19 patients treated with tocilizumab: an individual patient data systematic review. J Med Virol. 2020; 92:2516–22. https://doi.org/10.1002/jmv.26038 PMID:32436994

105. Azar KM, Shen Z, Romanelli RJ, Lockhart SH, Smits K, Robinson S, Brown S, Pressman AR. Disparities In Outcomes Among COVID-19 Patients In A Large Health Care System In California. Health Aff (Millwood), 2020: p. 101377hlthaff202000598.

106. Guo X, Zhu Y, Hong Y. Decreased Mortality of COVID-19 With Renin-Angiotensin-Aldosterone System Inhibitors Therapy in Patients With Hypertension: A Meta-Analysis. Hypertension. 2020; 76:e13–14. https://doi.org/10.1161/HYPERTENSIONAHA.120.15572 PMID:32458694

107. Shi L, Wang Y, Wang Y, Duan G, Yang H. Dyspnea rather than fever is a risk factor for predicting mortality in patients with COVID-19. J Infect. 2020; 81:647–79. https://doi.org/10.1016/j.jinf.2020.05.013 PMID:32417316

108. Singh AK, Khunti K. Assessment of risk, severity, mortality, glycemic control and antidiabetic agents in patients with diabetes and COVID-19: A narrative review. Diabetes Res Clin Pract. 2020; 165:108266. https://doi.org/10.1016/j.diabres.2020.108266 PMID:32533989

109. Ufuk F, Savaş R. Chest CT features of the novel coronavirus disease (COVID-19). Turk J Med Sci. 2020; 50:664–78. https://doi.org/10.3906/sag-2004-331 PMID:32394687

110. Ahmed SI, Hasan SM, Ahmed T. Obesity is a potential risk factor for covid-19 associated morbidity and mortality in urban Bangladesh. BMJ. 2020; 370:m2811. https://doi.org/10.1136/bmj.m2811 PMID:32665220

111. Chanana N, Palmo T, Sharma K, Kumar R, Graham BB, Pasha Q. Sex-derived attributes contributing to SARS-CoV-2 mortality. Am J Physiol Endocrinol Metab. 2020; 319:E562–67. https://doi.org/10.1152/ajpendo.00295.2020 PMID:32726128

112. Curigliano G. Cancer Patients and Risk of Mortality for COVID-19. Cancer Cell. 2020; 38:161–63. https://doi.org/10.1016/j.ccell.2020.07.006 PMID:32710820

113. de Sousa E, Ligeiro D, Lérias JR, Zhang C, Agrati C, Osman M, El-Kafrawy SA, Azhar EI, Ippolito G, Wang FS, Zumla A, Maeurer M. Mortality in COVID-19 disease patients: correlating the association of major histocompatibility complex (MHC) with severe acute respiratory syndrome 2 (SARS-CoV-2) variants. Int J Infect Dis. 2020; 98:454–59. https://doi.org/10.1016/j.ijid.2020.07.016 PMID:32693089
